# A Beam Hopping Scheme Based on Adaptive Beam Radius for LEO Satellites

**DOI:** 10.3390/s24206574

**Published:** 2024-10-12

**Authors:** Jinhui Chen, Quanjiang Jiang, Mubiao Yan

**Affiliations:** 1 Innovation Academy for Microsatellites of Chinese Academy of Sciences, Shanghai 201304, China; 2 University of Chinese Academy of Sciences, Beijing 100049, China

**Keywords:** SAGIN, LEO satellite communication, beam-hopping, resource allocation

## Abstract

Toward the vision of seamless global connectivity in the 6G era, the non-terrestrial network (NTN) in space-air-ground integrated networks (SAGINs) network architecture is one of the highly promising solutions. From the perspective of relay nodes, NTN includes satellite nodes and space-based platform nodes. As a resource management technology in satellite communication, beam-hopping has garnered significant attention from researchers due to its effectiveness in ad-dressing the disparity between offered capacities and uneven terrestrial traffic demands. Recognizing that the larger beams offer broader coverage but the smaller ones provide better an-ti-interference capabilities and higher throughput, this paper introduces an adaptive cluster-ing-based approach. It provides large, medium, and small user beams to target ground users. The proposed algorithm aims to minimize total system latency and enhance system throughput. Sim-ulation results show that employing the proposed algorithm in the baseline model results in a 3.44% increase in system throughput and a 35.5% reduction in system latency. Furthermore, simulation results based on alternative models indicate that while the proposed algorithm may lead to a slight decrease in system throughput, it brings significant improvements in system latency.

## 1. Introduction

The IMT-2030 (6G) Promotion Group released the White Paper on 6G Network Architecture Vision and Key Technology Outlook in September 2021. It outlines the development of future 6G network architecture, proposing that the 6G communication system should offer all-weather, three-dimensional network access services available worldwide [1,2] in the future. SAGINs is a key network architecture to realize the vision of ubiquitous 6G coverage, which comprises the terrestrial network and the NTN, consisting of space-based platforms and satellites. Of these, NTN achieves ubiquitous coverage in remote areas, and terrestrial networks realize regular coverage in non-remote areas [3]. As a key component of the NTN, LEO satellites are once again attracting the interest of re-searchers and industry. This paper will focus on radio resource management for LEO satellites.

Due to their limited payload capacity, current low Earth orbit (LEO) satellites are un-able to simultaneously serve all users within their field of view. The current approach to addressing this limitation involves providing services to as many users as possible through methods such as frequency division multiplexing, space division multiplexing, and time division multiplexing. The most promising method is beam-hopping technology originating from the resource management of the GEO satellites which has emerged as a hot topic in current research on LEO satellite communication.

Beam-hopping technology is a resource allocation technique, that is based on flexible devices for the dynamic allocation of communication resources between beams. Thus, this technique can better overcome the phenomenon of scarcity or excess of offered communication resources caused by uneven ground traffic distribution. Moreover, the efficient utilization of on-broad payloads can reduce the mass of satellites and the cost of launch.

## 2. Related Work

All kinds of advantages are driving researchers to research beam-hopping in any other aspects. Here is a summary of the current research on beam-hopping resource allocation: Currently, researchers model the beam-hopping problem as a convex or non-convex problem, depending on whether the inter-beam interference can be neglected or not. If the problem is a convex problem, a numerical solution can be obtained easily by solving the convex optimization problem; however, in practice, without interference isolation measures, co-frequency interference can result in significant bandwidth and power wast-age, leading to a severe degradation in system throughput. Therefore, the following meth-ods are commonly adopted to mitigate interference:

- Clustering beams to avoid co-interferences intra beam [4,5,6].
- Setting the interference distance as a guideline to avoid co-interferences within the intra beam [7,8].
- Adopting some algorithm to cancel interferences [9].
- Adopting the algorithm that is driven by data [10,11,12,13,14].

Among the aforementioned interference avoidance methods, there are many different approaches, each with distinct implementation details. Taking the commonly practiced method of setting interference avoidance thresholds as an example, we conduct analysis and discussion: Lingkai Zhao [7] used distance as the independent variable to simulate and visualize the relationship between signal-to-noise ratio (SNR) and signal-to-interference-plus-noise ratio (SINR). Based on this simulation, the authors identified the distance at which SINR is approximately equal to SNR and determined a threshold distance at which interference from co-channel beams can be neglected. This is a feasible method for interference avoidance, and this approach will be continued in this paper, with relevant discussions placed in Section 3.6.

On the one hand, this paper adopts interference avoidance strategies from related works, while on the other hand, it identifies the lack of utilization of beam flexibility in previous works. Therefore, building upon prior research, this paper proposes a communication strategy that utilizes beams of three different radii to serve ground users. The following is a summary and analysis of previous works:

In recent years, researchers have gradually realized the beam flexibility thanks to the advancement of phase array antennas and the computing capability on board, and some papers have begun to study the use of phased array antennas based on their flexible beam pointing and the ability to dynamically change the beam radius to fully serve terrestrial users. Tang et al. [15,16] used the P-Center algorithm to determine the beam center and radius based on the user distribution, which is difficult to realize in practice on one hand, and on the other hand, when the number of users in the non-hot spot area is small, the determination of the beam center and radius only relying on the user distribution leads to unreasonable distribution. Guoliang Xu et al. [17] proposed an iterative algorithm and reinforcement learning to allocate resources to users, although both of these algorithms have very high computational complexity. Leyi Lyu et al. [18] found the sparsest user by the user location matrix and user density vector, and then gradually expanded the radius to accommodate more users, until the beam could not meet the users’ demands or all the users had been assigned to beams. This algorithm belongs to the iterative algorithm, which has a large time and space complexity. Chunge Xu [19] gave a simple and easy beam scheduling method by comprehensively considering the user traffic demand and distribution, but the method does not consider the system delay, which plays an important role in user quality of service (QoS). Tao Zhang et al. [20] defined resource utilization rate in their article first, then adjusted beam radius to make resource utilization rate tend to 100% in a simplified scene, but the author did not consider the result caused by expanding the beam radius that directly leads to intra-beam interference. Lei L et al. [21] first proposed a simplified scenario, then conducted an analysis based on this scenario in their study. Then, they concluded that satellites should provide narrow beam coverage while also prioritizing users with high channel gain and traffic demand to achieve maximum throughput. Based on the above conclusions, the authors proposed an algorithm that adjusts beam radius and allocation beam position based on the indicators $\mu_{sk}$ and $v_{sn}$ that depend on users’ gain and traffic demand, and location. The algorithm has a strong physical significance, but it ignores users’ QoS and consumes so many computational resources in the background that its on-broad computational capability is limited.

On the foundation of previous research, we take the system throughput and total system delay into account and utilize the features of large size beams’ coverage capability, and small size beams’ interference avoidance capability, high gain in this article. Furthermore, we allocate the three kinds of beams to suitable positions at the correct time so that terrestrial users can enjoy better service quality and achieve system performance improvement. The simulation result shows that employing the proposed algorithm in the baseline model results in a 3.44% increase in system throughput and a 35.5% reduction in system latency. Moreover, in alternative models, the proposed algorithm consistently enhances system performance.

The remaining chapters of the article are organized as follows: Chapter 3 focuses on proposed required system parameters and system modeling, which constructs the model required by simulation; Chapter 4 contains a simulation scene and algorithm introduction, four simulations to examine the proposed algorithm in four aspects are conducted; in addition, the relevant simulation results are presented in visualized form and analyzed in detail; and Chapter 5 summarizes this article.

## 3. Model

This section may be divided by subheadings. It should provide a concise and precise description of the experimental results, their interpretation as well as the experimental conclusions that can be drawn.

### 3.1. Traditional Beam-Hoppping Model

Considering a high throughput multi-beam satellite system with $N_{B}$ beams. In order to adapt to the changing traffic distribution and the increasing demand for Internet traffic, this paper adapts the on-demand resource allocation and full frequency reuse strategies. To provide on-demand services more flexibly for users, we allow beams to hop within the field of view under the constraint of interference angle. In addition, we focus on the gateway-satellite-user information stream of a broadband multi-beam satellite system.

**Figure 1:**
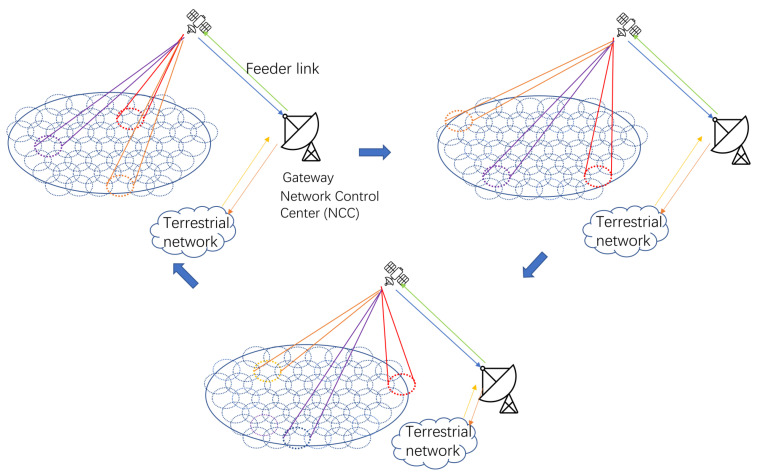
Beam-Hopping working procedure.

The beam hopping model based on the above considerations is shown in Figure 1.

In the Figure 1, we can see that: according to the beam hopping time plan (BHTP) formulated by the ground network control center (NCC), the on-board computer guides the beam to point to the cell planned to be served in the schedule; meanwhile, a portion of the data packets arriving at the gateway station are transmitted to the destination terminals via the forward link, while the remaining portion is temporarily buffered in the message cache queue at the gateway station. After the end of the current time slot, the on-board computer will control the beam to point to the next cell to be served according to the BHTP, and so on.

In this article, we define $N_{B}$ as the number of user beams, which is the channel of line of sight communication, $N_{cell}$ is the number of cells in the satellites’ field of view. The hopping windows is $T_{H}$ which is equally divided into $N_{T}$ slots, a time slot is $T_{slot}=\frac{N_{B}}{N_{T}}$ long in time. In each of time slots, NCC should allocate power to each cell. The power that $cell_{i}$ acquires in time slot t denoted as $P_{cell_{i},t}$.

Randomly distributed users randomly generate service demands, and the length of the data packet whose destination user is $user_{i}$ is denoted as “$pck^{user_{i}}.len$” The arriving time of the data packet is “$pck^{user_{j}}.{start}_{time}$”. Similarly, the leaving time of the data packet is $pck^{user_{j}}.{end}_{time}$. The delay of the above packet is denoted as *D* equals to $pck^{user_{j}}.{end}_{time}-pck^{user_{j}}.{start}_{time}$. Moreover, the total delay of $cell_{i}$ is $D_{cell_{i}}$ which equals to $D_{cell_{i}}=\sum_{pck\in cell_{i}}D$. The system delay is $Delay=\sum_{i=0}^{N_{cell}}D_{cell_{i}}$.

The total service demand of the $user_{j}$ in the kth time slot is defined as $D_{user_{j},k}$, and the throughput provided by the communication system for the user is $R_{user_{j},k}$. The service demand of $cell_{i}$ is $D_{t}^{cell_{i}}$ equaling to $\sum_{j}D_{user_{j},t}$, the offered throughput to $cell_{i}$ is $R_{t}^{cell_{i}}$, To avoid wasting communication resources, the actual rate provided should be equal to the cell’s bandwidth demand. Therefore, actual throughput is used to measure the degree of service satisfaction for service cells. The actual throughput, denoted as Throughput, is equal to $\sum_{t=0}^{99}\;\sum_{i=0}^{N_{cell}}\;min\left(D_{t}^{cell_{i}},R_{t}^{cell_{i}}\right)$. It is worth noting that the effective throughput Throughput is obtained by summing the effective throughput of all service cells across all time slots.

The delay sensitivity factor is ρ, and the virtual demand traffic of a packet is:

$pck^{user_{i}}.len\times \rho^{pck^{user_{i}}.\mathrm{end}\_\mathrm{time}-pck^{user_{i}}.\mathrm{start}\_\mathrm{time}\;}$

and the virtual demand traffic of $cell_{i}$ is:

$VirD_{t}^{cell_{i}}=\sum_{pck\in cell_{i}}pck^{user_{i}}.len\times \rho^{t-pck^{user_{i}}.{\mathrm{start}}_{t}\mathrm{ime}}$.

We introduce virtual traffic demand that not only considers the significance of broadband packets but also takes into account the importance of delay packets that are measured by ρ and the time to live. Both of them guarantee the users’ QoS by participating in cell selection. The above parameters are somewhat complex. Table 1 below provides a clear presentation of the symbols and definitions of these parameters for better understanding.

### 3.2. System Parameter

Table 2 presents the parameters used in the simulation. Additionally, these parameters are also provided in tabular form for ease of reading and reference by others.

### 3.3. Transport Model

Channel coefficients:

(1) $$h_{k,n}=\frac{\sqrt{G_{R}G_{k,n}}}{4\pi \frac{d_{k}}{\lambda }\sqrt{K_{B}TB}},$$

$h_{k,n}$ is the channel coefficient of the link between $user_{k}$ and beam *n*. $G_{R}$ refers to the receiving antenna gain of the receiving terminal, $G_{k,n}$ refers to the transmitting antenna gain between $user_{k}$ and beam *n*, $d_{k}$ refers to the distance between the user and the satellite, *T* refers to the noise temperature of the terminal, *B* refers to the bandwidth, and $K_{B}$ refers to the Boltzmann constant.

A model with signals that have undergone large-scale fading channels and have been added with Gaussian white noise:

(2) $$y_{k,n}=h_{k,n}^{T}x+n_{k,n},$$

x is the transmitted signal vector, and $n_{k,n}$ is the additive Gaussian white noise.

$SINR_{k}$:

(3) $${\mathrm{SINR}}_{k}=\frac{|h_{k,n}|\cdot P_{n}}{\sigma^{2}+\sum_{m\neq n,j\neq k}|h_{j,m}|\cdot P_{m}}$$

$SINR_{k}$ is the signal- to- interference plus noise ratio suffered by $user_{k}$. The above formula represents the SINR equation in the manuscript. In this equation, there is one term in the numerator and two terms in the denominator. The numerator term is the product of the magnitude of the complex channel coefficient and the power allocated to $user_{k}$. This term represents the energy of the useful signal.

Let us now consider the denominator. $\sigma^{2}$ is the noise signal energy. As long as the temperature of an object is above absolute zero, thermal noise will be present. $\sum_{m\neq n,j\neq k}|h_{j,m}|\cdot P_{m}$ represents the interference signals energy. From the expression of this term, it appears to be the summation of the energy of many useful signals. However, since these useful signals are intended for users other than $user_{k}$, they consequently interfere with $user_{k}$. By summing up these interfering signals, we can obtain the total energy of the interference signals.

The traditional solution to reducing interference is keeping sufficient distance be-tween service beams to reduce interfering antenna gain and deploying spot beams.

$SNR_{k}$:

(4) $$SNR_{k}=\frac{\left|h_{k,n}\right|\cdot P_{n}}{\sigma^{2}}$$

Shannon formula:

(5) $$R_{k}^{t}=B_{k}^{t}\times log_{2}\left(1+SINR_{k}^{t}\right)$$

$B_{k}^{t}$ represents the bandwidth allocated to $user_{k}$ by the satellite at time *t*. $R_{k}^{t}$ is the information rate of $user_{k}$ at time *t*.

Offered throughput in $cell_{i}$:

(6) $$SNR_{k}=\frac{\left|h_{k,n}\right|\cdot P_{n}}{\sigma^{2}}$$

### 3.4. Cell Model

We calculate the middle beams’ radius, which is the baseline, to determine the largest size beam and the smallest beam.

Assuming the user is in the coverage area of a user beam, the users’ minimum elevation angle is 85°, denoted as *E*, and then the, Earth Central Angle α can be calculated by equation:

(7) $$\alpha =\arccos\left(\frac{R_{E}\cos E}{R_{E}+h}\right)-E$$

The area covered by the middle-sized beam is:

(8) $$A_{S}=4\pi {R_{E}}^{2}\sin^{2}\frac{\alpha }{2}$$

The radius of that can be derived from the equation:

(9) $$r=\sqrt[{2}]{\frac{A_{S}}{\pi }}$$

### 3.5. Antenna Model [22]

Table 3 illustrates the relationship between coverage radius and antenna gain. As shown in the table, beams with a smaller coverage radius exhibit a higher concentration of energy and consequently greater antenna gain, while the opposite is true for larger coverage radii.

The normalized antenna gain calculation formula is:

(10) $$G\left(\theta \right)=10\times \mathrm{lg}(|\frac{\sin(N\times \pi \times \sin\left(\frac{\theta }{2}\right))}{N\times \sin(\pi \times \sin\left(\frac{\theta }{2}\right))}|)$$

**Figure 2:**
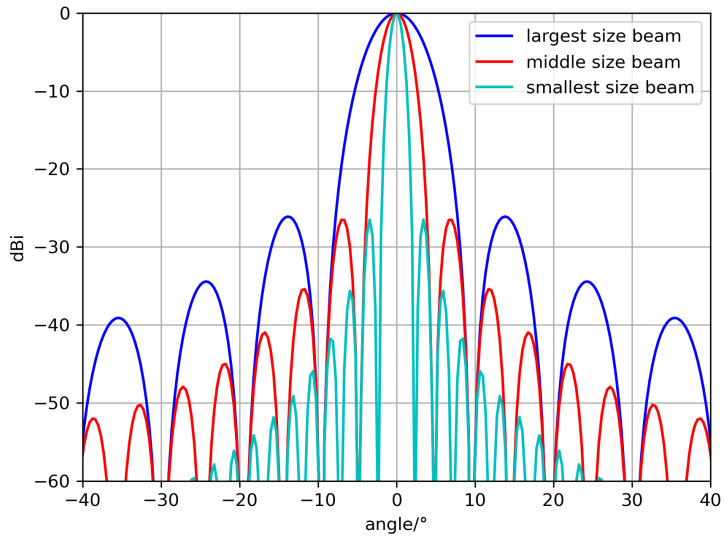
The normalized radiation pattern of three sizes of beams.

Based on Equation (10), the antenna radiation patterns of the three beams are visualized, and the corresponding results are presented in Figure 2.

### 3.6. Interference Avoid Model [7]

According to the SNR formula, the interference power can be considered negligible when the noise power is much larger than the interference power.

The Formula (4) can be expand to:

(11) $$SNR_{k}=\frac{\left|h_{k,n}\right|\cdot P_{n}}{\sigma^{2}}=\frac{P_{t}\times G_{t}\times G_{r}}{Loss\times k\times T\times B}$$

$\frac{P_{t}\times G_{t}\times G_{r}}{Loss}$ is the magnitude of the complex channel coefficient.

Formula (3) can be expanded to:

(12) $$SINR_{k}=\frac{\frac{P_{t}\times G_{t}\times G_{r}}{Loss}}{\left(k\times T\times B+\frac{P_{t}\times G_{t}\left(\theta \right)\times G_{r}}{Loss}\right)}$$

Considering $d=\frac{\lambda }{2}$, the antenna array factor can be deduced:

(13) $$G_{t}\left(\theta \right)=\frac{\sin\left(\frac{N\pi }{2}\sin\left(\theta \right)\right)}{Nsin\left(\frac{\pi }{2}\sin\left(\theta \right)\right)}$$

When $k\times T\times B\gg \frac{P_{t}\times G_{t}\left(\theta \right)\times G_{r}}{Loss}$, the interference power can be neglected, and considering the above equations, the interference avoidance angle can be solved:

(14) $$\hat{\theta }=G_{t}^{-1}\left(\frac{k\times T\times B\times \;\mathrm{Loss}\;}{P_{t}\times G_{r}}\right)$$

**Figure 3:**
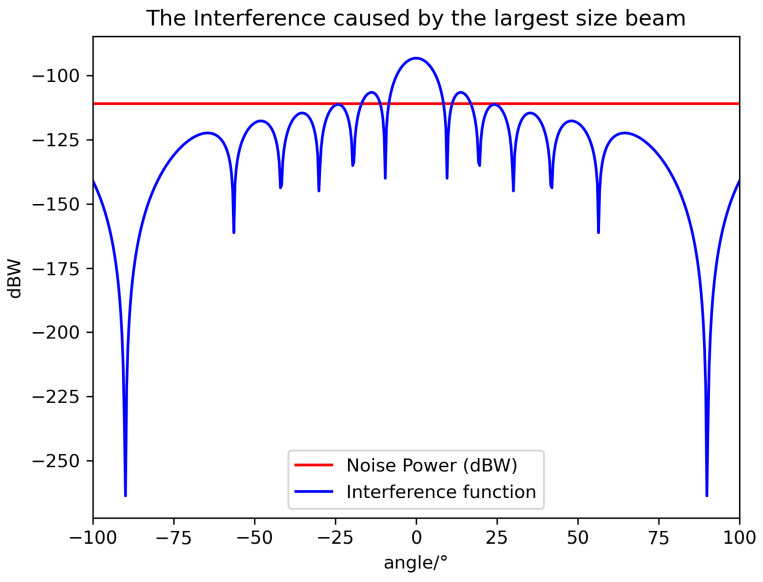
The relationship between noise power and interference caused by the largest-sized beam.

**Figure 4:**
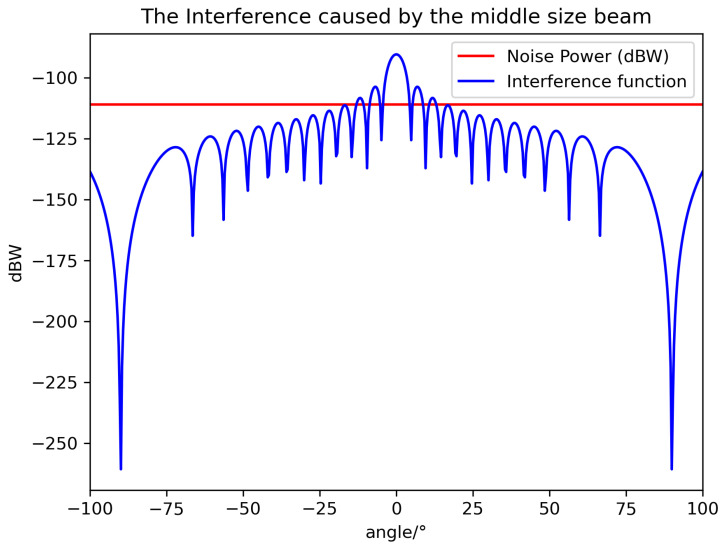
The relationship between noise power and interference caused by a middle-sized beam.

**Figure 5:**
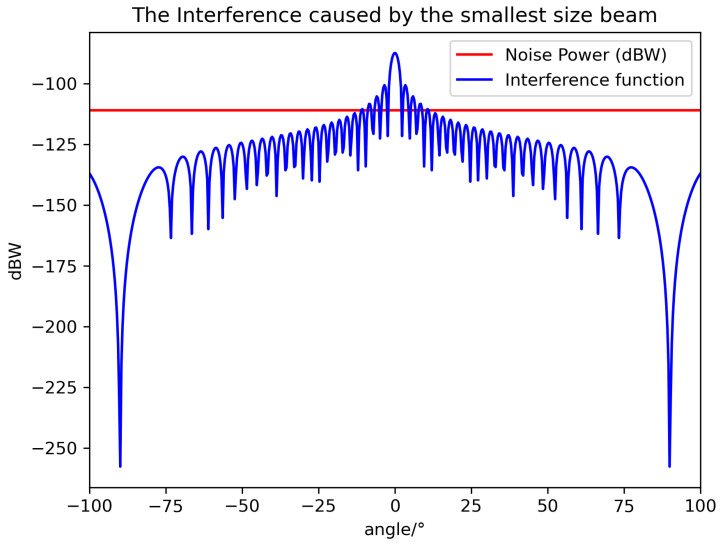
The relationship between noise power and the interference caused by the smallest-sized beam.

It is worth noting that $\hat{\theta }$ is the minimum lower bound, and θ should be larger than that to avoid stronger interference. According to the above equation and system value, we can visualize the value relationship between noise power and interference power in Figure 3, Figure 4 and Figure 5.

We can infer from Figure 3 that the largest beams can provide the largest coverage for users to access the Internet, but on the other hand, it they will lead to stronger interference than other beams because their gain drops slowly. Therefore, the placement of the largest beam is the first kind of beam to take into account; otherwise, the users’ Qos can not be guaranteed. In addition, the position and the time at which the largest beam is placed are the most difficult problems that we will address in this article.

It can be seen that the smallest beam has the smallest off axis angle to avoid interference thanks to its fast-decaying gain curve. It is not easy to cause interference; the reverse is true as well. The experimental results are in line with existing research findings.

### 3.7. User Distribution Model [23]

**Figure 6:**
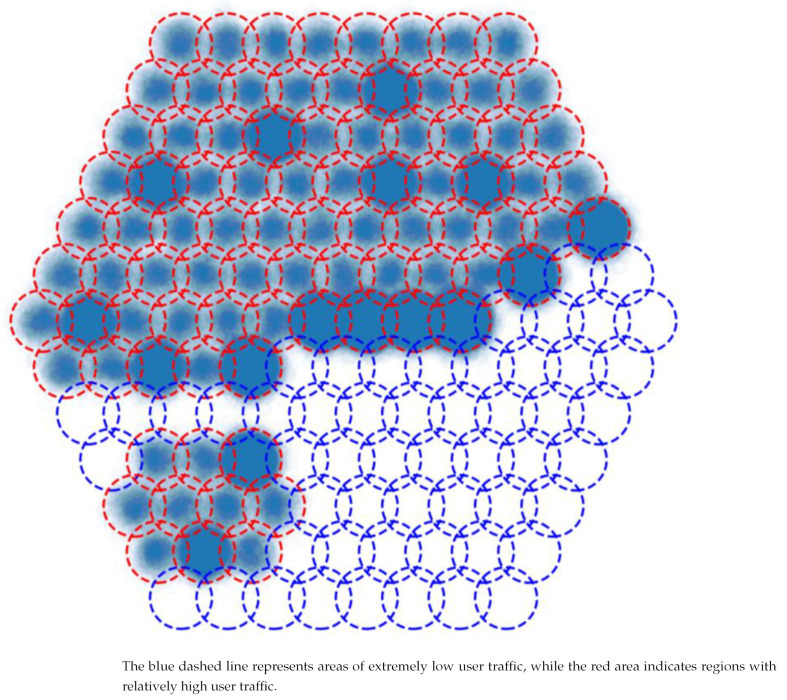
Traffic distribution.

Depending on Satellite Image of Urban Night Lights, we generate the following traffic distribution to launch the simulation. In the Figure 6, the bluer the color, the higher the number of users. The red and blue dashed lines represent the virtual cell boundaries over land and sea, respectively.

We have determined the distribution of users based on the above graph, but we still need to determine the traffic per user. In this article, we set user broadband services packets to follow a standard normal distribution with μ = 15 and σ = 5, on the other hand, we set user delay services packets to follow a uniform distribution with μ = 1.

### 3.8. Problem Formulation

Based on the aforementioned model, we first present the problem that this paper aims to address, followed by a mathematical formulation of the issue.

From the aforementioned model, we observe that the number of concurrently operating beams is limited, and the power provided by the amplifiers for each beam is also constrained. Additionally, to avoid interference between beams, it is necessary to maintain a specific isolation angle. Therefore, we select the cell with the maximum value of A from those that meet these requirements.

Below, I use mathematical expressions to represent these constraints and my optimization objectives.

$$\begin{array}{c} P1:\max Throughput\\ P2:\min Delay\\ \;\;s.t.\\ C1:0\leq P_{cell}^{i}\leq P_{max}\\ C2:0\leq \left|C\right|\leq N_{\mathrm{beam}}\\ C3:i=\mathrm{argmax}\left(VirD_{t}^{cell}\right)\\ C4:<cell_{i},cell_{j}>\geq \theta_{\mathrm{th}}\left(cell_{j}\in C\right) \end{array}$$

In the above problem, *C*1 and *C*2 means power and bandwidth must not exceed the maximum power and total bandwidth of the transponder. *C*3 indicates the cells are chosen by its max $VirD_{t}^{cell_{i}}$ value; we choose the biggest $N_{beam}$$VirD_{t}^{cell_{i}}$ value under the constraint of *C*4. *C*4 constrains the angle between the $cell_{i}$ and the cells which is chosen completely. In conclusion, *C*3–*C*4 means we can choose up to $N_{beam}$ cells from $N_{cell}$ cells that should have enough $VirD_{t}^{cell_{i}}$ and $cell_{i}$ should satisfy interference avoid criterion from *C*4.

### 3.9. Proposed Algorithm Model

**Figure 7:**
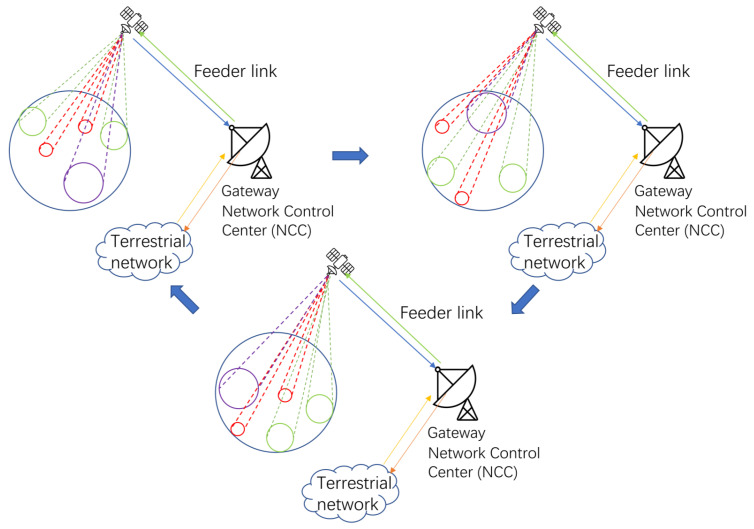
Proposed beam hopping working procedure.

We can see three sizes of beams to provide an equal number of services to different users from Figure 7. It is worth noting that beam should stay far apart to avoid interference in the full-frequency reuse scene.

### 3.10. Proposed Algorithmic Process

The following presents the algorithmic procedure of Algorithm 1.
**Algorithm 1** This algorithm takes the virtual traffic of each cell as input and generates a BHTP based on the templates prepared during the initialization phase  1:**Initialization:** A=∅, $\Phi_{1}=\Phi_{2}=\ldots =\Phi_{k}=\ldots =\Phi_{K}=\emptyset$, R=∅, $\phi_{1}$, $\phi_{2}$, *…*, $\phi_{K}$  2:**Input:** $\;Traffic_{beam,i}^{t},K=4$  3:**Step 1: Choose non-hotspot cells to form A**  4:**for** $\;Traffic_{large,i}^{t}=1,2,\ldots ,M\;$ **do**  5:     **if** $Traffic_{large,i}^{t}\leq Traffic_{threshold}^{t}$ **then**  6:         Add $cell_{i}$ into A  7:**Step 2: Partition non-hotspot cells into K clusters by $\phi_{k}$, ∀k=1,2,…,K**  8:**for** each element $e_{i}$ in A **do**  9:     **if** $e_{i}\in \phi_{k},\forall k=1,2,\ldots ,K$ **then**10:         Add $e_{i}$ into the $\Phi_{k}$11:Assign K consecutive time slots to serve the above K clusters, with one time slot for each cluster.12:**repeat**13:     **if** there are speared beams **then**14:         **Step 3: Choose medium and small cells to the clusters served in current time slots**15:         **for** $Traffic_{middle,i}^{t}=1,2,\ldots ,N$ **do**16:            **if** $cell_{i}$ has no interference with chosen cells and $Traffic_{middle,i}^{t}$ >  threshold **then**17:                $cell_{i}$ will be served as a medium cell in current time slot.18:         **if** there are speared beams **then**19:            **for** $Traffic_{small,i}^{t}=1,2,\ldots ,N$ **do**20:                **if** $cell_{i}$ has no interference with chosen cells **then**21:                    $cell_{i}$ will be served as a small cell in current time slot.22:**until** end of simulation

### 3.11. Computational Complexity Analysis

In step 1, we find the non-hotspot cell by traversing the macro cell service traffic. Therefore, the algorithmic computational complexity of step 1 is O(M), *M* is the number of large cells.

In step 2, we partition *M* cells into four clusters, thus, the algorithm’s computational complexity is O(M).

In step 3, if there are available service beams, they will be utilized to serve medium or micro cells. Therefore, the algorithm complexity is O(N∗I)), where *N* is the number of medium cells and *I* is the number of micro cells.

In general, due to the significantly smaller number of large-beam cells compared to medium- and small-beam cells within the same coverage area, the product of *N* and *I* is much larger than *M*. Therefore, the algorithm’s computational complexity is denoted as O(N∗I).

## 4. Presentation and Analysis of Simulation Results

In this chapter, we will list all of our simulations that aim at examining the proposed algorithm in different aspects that can cause different system performance, and then visualize the results. At the end of every simulation, we will analyze and explain the results

### 4.1. Comparison of Proposed and Traditional Algorithm in Constructed Scene That Defined in User Distribution Model

**Figure 8:**
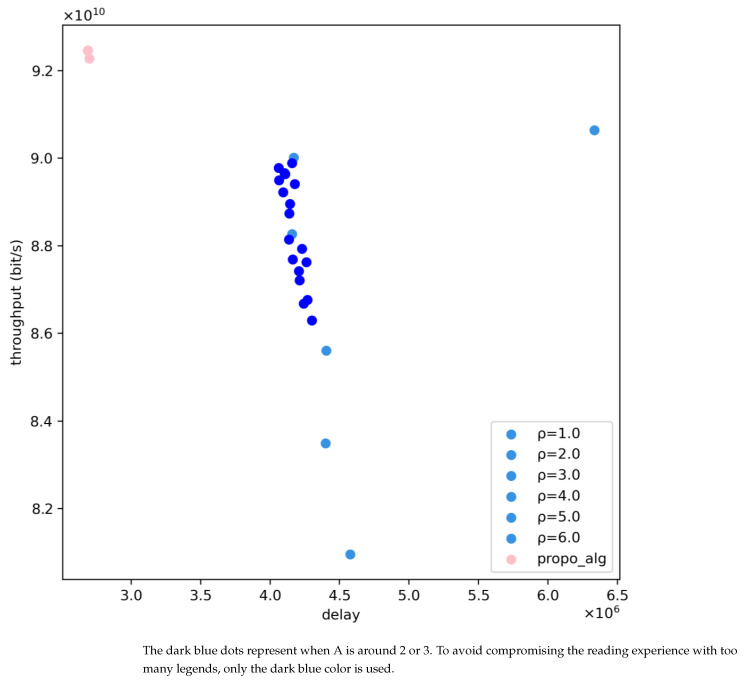
Comparison of total delay and throughput between the proposed and traditional algorithms in the constructed Scenario.

In this simulation, we make a comparison between the traditional and proposed algorithms in the model constructed in Section 3.7. In addition, we change the delay sensitivity factor that is beneficial to reduce system delay to examine the proposed and traditional algorithm’s performance. Figure 8 illustrates the performance of the two algorithms based on the constructed model. The pink dots represent the system performance metrics obtained by the proposed algorithm, while the blue dots represent the system performance metrics obtained by the traditional algorithm. It can be observed that with the adoption of the proposed algorithm, there is a 3.44% improvement in system throughput, and the system delay is reduced by 35.5% compared to a delay factor of 2. Based on these results, further experiments were conducted to reduce system delay by increasing the delay sensitivity factor and examining the loss of system throughput.

According to the experimental data, it is observed that when the delay sensitivity factor is 1, the system delay is reduced by 58.54%, and throughput is increased by 2.77%. When the delay sensitivity factor is 3, the system delay is reduced by 35.29%, and throughput is increased by 5.31%. When the delay sensitivity factor is 4, the system delay is reduced by 38.93%, and throughput is increased by 8.17%.

**Figure 9:**
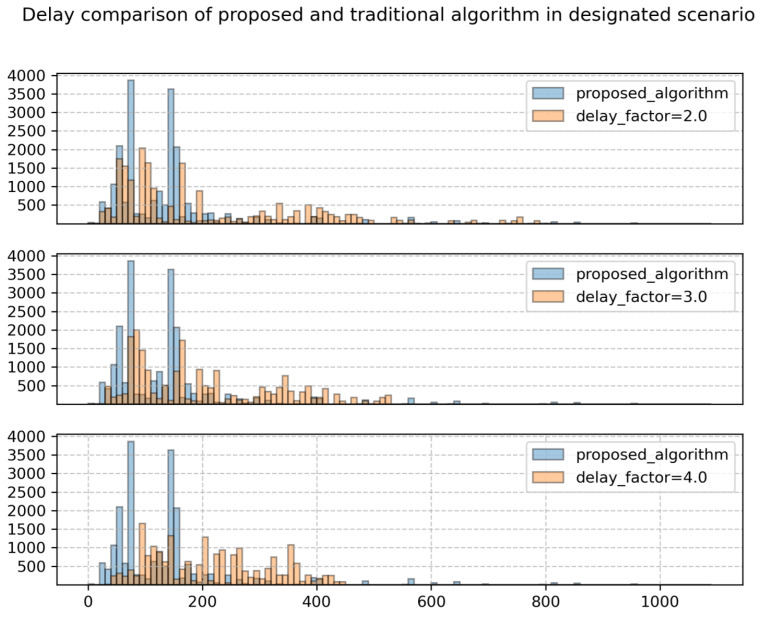
The distribution of delay for all delay users.

Figure 9 depicts the delay distribution of all delay users under three delay factor scenarios. It can be observed that when the delay sensitivity factor is 2, the delay distribution range of delay users is very wide, ranging from 0 to 800. However, when the simulation adjusts the delay factor to 5, the delay distribution range of delay users is narrowed, with no users having a delay greater than 600. However, the number of low-delay users also decreases. This indicates that the increase in the delay factor sacrifices the QoS of hot spot area users while improving the QoS of non-hot spot area users, especially those adjacent to hot spot areas. When the delay factor is increased to 4, the delay distribution range is further compressed. However, the performance improvement in delay at this point is not as significant as it was when the delay factor increased from 2 to 3. This is because the satellite cannot serve all users at all times. Under the current service conditions, there is inevitably a minimum total delay greater than 0, and it becomes increasingly difficult to improve the delay performance as it approaches this value.

Furthermore, in the three subplots of Figure 9, the blue bars represent the distribution of user delays after applying the proposed algorithm, while the orange bars represent the distribution of user delays based on the traditional algorithm with different delay sensitivity factors. From the distribution of the blue bars compared to the orange bars, it can be inferred that the proposed algorithm, compared to the traditional algorithm, can reduce the number of high-delay users, thereby reducing the total system delay.

### 4.2. Comparison of Proposed and Traditional Algorithms in the Strongest Interference Scene

In this simulation, we constructed a different traffic model with the strongest interference to test the proposed and traditional algorithms performance.

**Figure 10:**
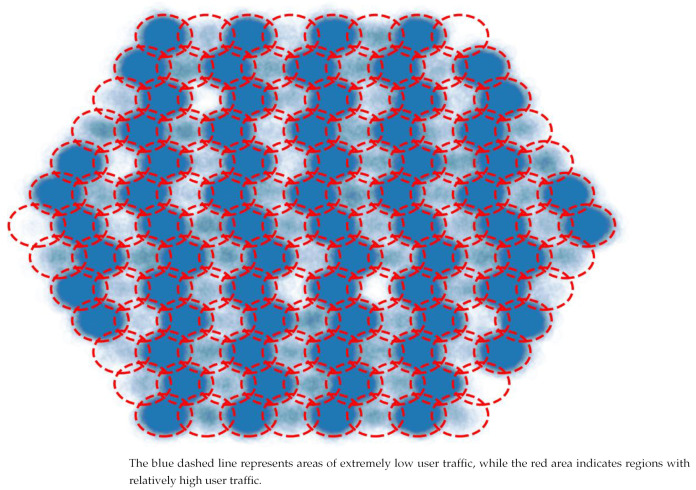
The constructed traffic distribution.

We constructed the traffic distribution with the strongest interference so that the hot spot area and non-hot spot area are alternated to examine the proposed and traditional algorithms’ performance. The distribution is shown in Figure 10.

**Figure 11:**
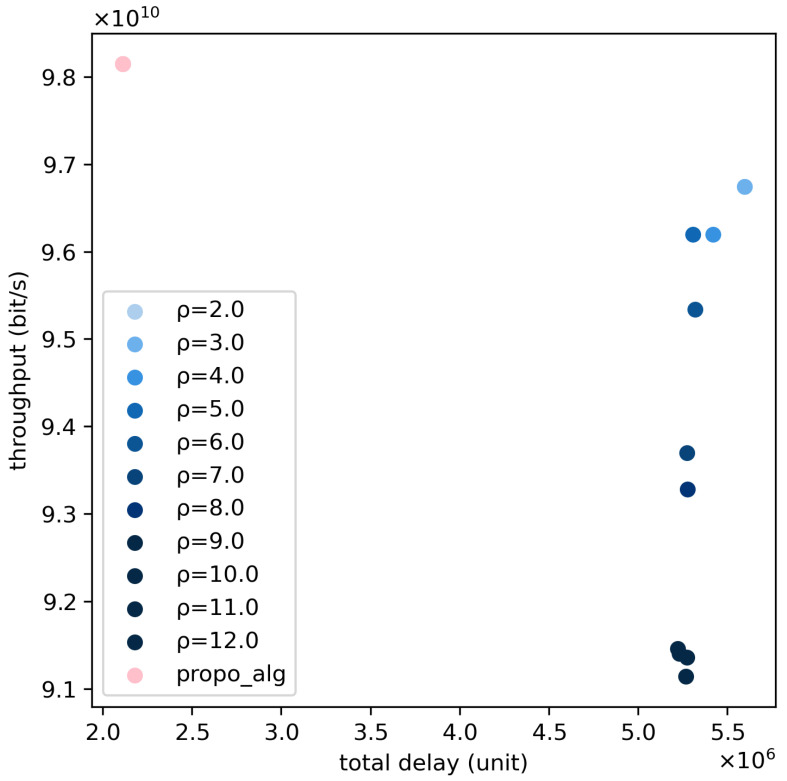
The comparison of total delay and throughput between proposed and traditional algorithms in the new scenario.

**Figure 12:**
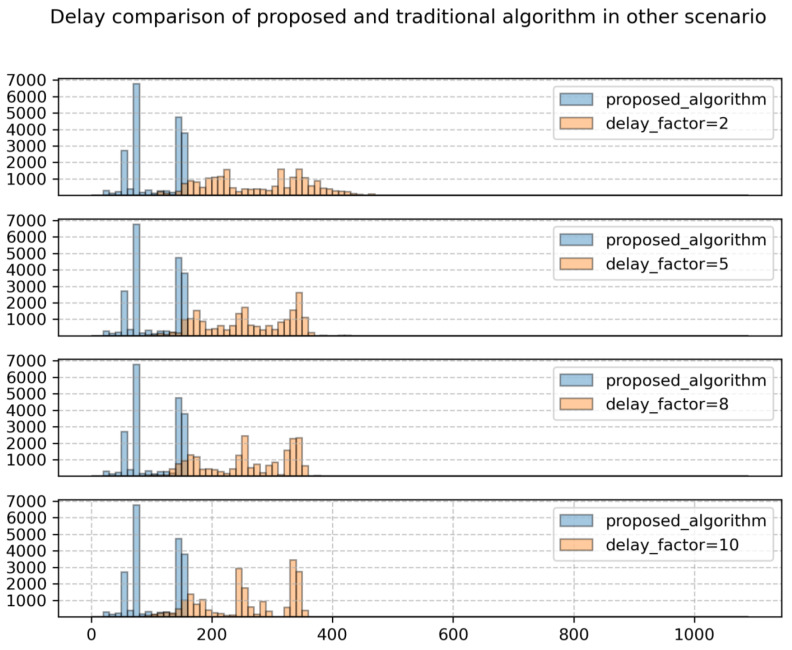
Users delay distribution.

Figure 11 illustrates the comparison of system performance between the proposed algorithm and the traditional algorithm under the distribution of strong potential interference services. It can be observed that the system throughput and total system delay of the system based on the proposed algorithm outperform those of the traditional algorithm. Specifically, compared to the traditional algorithm with a delay sensitivity factor of 2, the proposed algorithm achieves a 1.43% increase in total system throughput and a 62.25% improvement in system delay optimization. This is because the proposed algorithm reasonably utilizes the broad coverage characteristics of large beams to serve ground users frequently, significantly reducing ground user delays. From the user delay distribution shown in Figure 12, it can be clearly seen that after adopting the proposed algorithm, the number of low-delay users is much higher than that of the traditional algorithm, while the number of high-delay users is much lower than that of the traditional algorithm.

In addition, this simulation examines the system performance of the traditional algorithm using different delay sensitivity factors. It can be observed that, similar to Figure 9, the system performance of the traditional algorithm still follows the trend of smaller delays and lower throughput as the delay factor increases. However, when the delay sensitivity factor reaches a certain level, the system throughput decreases rapidly, while the reduction in total system delay is not significant. This is because the system is already close to the minimum delay, so increasing the delay sensitivity factor to serve more non-hotspot area users results in a limited reduction in delay. Moreover, due to the interlaced distribution of non-hotspot and hotspot areas in the set scenario, the influence between adjacent cells be-comes stronger. Adopting a larger delay sensitivity factor will reduce the service time for more hotspot area users around the cells, leading to a significant decrease in throughput, resulting in a cliff-like deterioration in throughput when the delay sensitivity factor equals 5.

From Figure 12, it can be observed that, similar to Figure 9, increasing the user delay sensitivity factor results in a decrease in the number of high-delay users. However, unlike Figure 9, there is no noticeable reduction in the number of low-delay users here. Therefore, increasing the user delay sensitivity factor at this point will not lead to system delay deterioration as seen in Figure 9, but it will also result in a decrease in system throughput.

### 4.3. Comparison of Proposed and Traditional Algorithms with an Increasing Number of Beams

This simulation is the most interesting one, in my opinion, because it investigated the relationship between overall system throughput and delay with respect to the number of beams, under the traffic distribution introduced in Section 3.7. We still adopt this traffic model in the remaining simulation.

**Figure 13:**
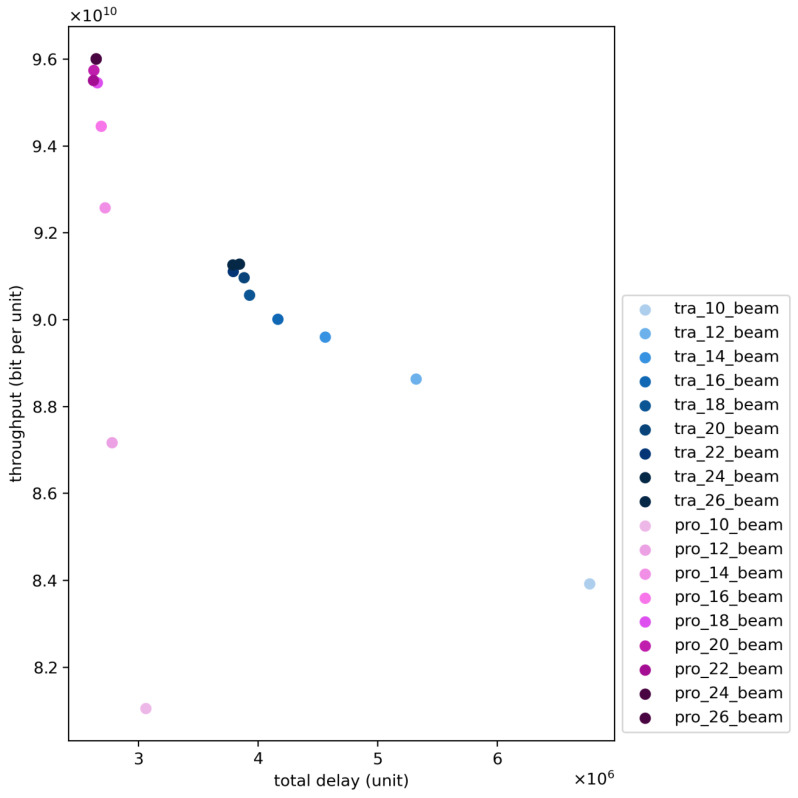
Comparison of the proposed and traditional algorithms with an increasing number of beams.

Figure 13 illustrates the variation in system performance based on the proposed algorithm and the traditional algorithm as the number of beams changes from 10 to 26. It can be observed that as the number of beams increases to 16, there is a certain improvement in system performance, but further increase in the number of beams leads to a bottleneck in system performance due to potential interference.

Furthermore, it can be noted that with the increase in the number of beams, the improvement in system performance due to beam addition shows a trend of diminishing re-turns, termed as “diminishing returns”, which is attributed to the increasing restrictions on beam utilization and the resulting potential interference. When the potential interference becomes significant, the addition of beams does not enhance system performance.

Additionally, by comparing the traditional algorithm with the proposed algorithm, it is evident that when the number of beams is relatively small, the traditional algorithm achieves a maximum performance improvement of approximately 5.19% in system throughput, attributed to the weaker service capability of large beams compared to traditional beams. However, as the number of beams increases, the proposed algorithm achieves superior system throughput performance. Moreover, under any number of beams scenarios, the proposed algorithm achieves at least a 30% improvement in delay optimization.

### 4.4. Comparison of Proposed and Traditional Algorithms with an Increasing Population

In this simulation, we change each cell’s population from 0.6 times the population to 1.4 times the population to examine the gap between two kinds of algorithms.

**Figure 14:**
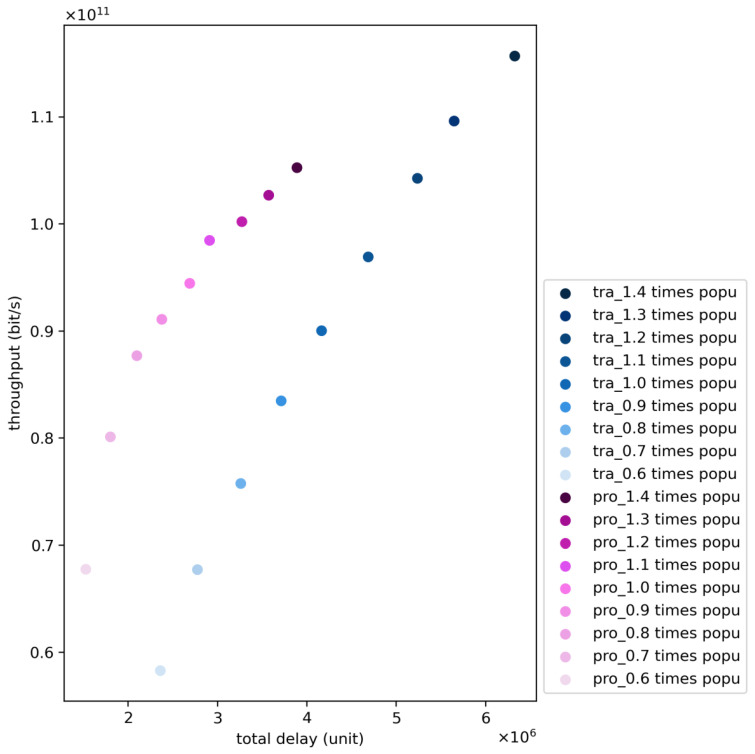
Comparison of the proposed and traditional algorithms with an increasing number of beams.

Figure 14 depicts the variation in system performance based on the proposed algorithm and the traditional algorithm as the number of users increases from 0.6 times to 1.4 times the original number. It can be observed that as the number of users increases, the system throughput also increases. This is because when calculating throughput, the actual throughput is considered, i.e., the minimum value between user demand and provided throughput. Therefore, when user demand increases, the system throughput either in-creases or remains unchanged. The increase in throughput indicates that the system capacity fully meets the total business requests of the benchmark user population; conversely, if the system throughput does not increase further, it suggests that the total business re-quests of the user population cannot be met. The simulation results in the figure also indicate that the increase in system throughput is not linear but slows down over time, influenced by the upper limit of system throughput.

Considering the variation in system delay, it can be observed that as the number of users increases, the total system delay also increases. This is because LEO broadband satellites are typical resource-constrained communication nodes with limited payload capacity. Therefore, they cannot serve all users simultaneously. When the number of users in-creases, the number of users served by LEO broadband satellites also increases, but at the same time, the number of unserved users also increases, leading to an overall increase in system delay.

Considering the performance variation of the algorithm with changes in the number of users, it can be seen that as the number of users increases, the gap between the system performance based on the proposed algorithm and that based on the traditional algorithm gradually widens. Specifically, as the number of users increases, the gap in system delay between the traditional algorithm and the proposed algorithm gradually increases. This is because with more users, the number of non-hotspot area users also increases, and the proposed algorithm is better able to ensure their QoS, thereby reducing the total system delay. On the other hand, considering the throughput of the proposed algorithm and the traditional algorithm, it is observed that as the number of users increases, under equal user conditions, the system throughput obtained by the proposed algorithm gradually decreases compared to that obtained by the traditional algorithm. This is because, on one hand, the proposed algorithm introduces wide beams, leading to stronger potential interference and reduced system throughput. On the other hand, as the number of users in-creases, the service demand of former non-hotspot areas exceeds the service capacity of large beams, making them hotspot areas. Limited by the weaker service capability of large beams, the system throughput cannot achieve the same level of system performance as the traditional algorithm.

## 5. Discussion

According to the simulation results, the proposed algorithm has both advantages and disadvantages. One drawback of adopting the proposed algorithm is that when the number of beams is insufficient or the number of users is too large, the overall system throughput compared to using traditional algorithms may decrease. This is because the proposed algorithm em-ploys wide beams with slow gain attenuation on the one hand, and on the other hand, to reduce computational complexity, it uses more wide beams, resulting in high potential in-terference and low transmission gain. Fortunately, due to thorough research before system deployment and the ease of deployment and technological upgrades of micro satellites in orbit, issues such as an excessive number of users and insufficient satellite beam numbers are generally less likely to occur. Nevertheless, for the algorithm to be more robust, I suggest that in the future, artificial intelligence algorithms such as deep learning can be used on the basis of this algorithm to intelligently utilize various types of beams.

The advantage of adopting the proposed algorithm is that it significantly reduces the overall system latency and slightly improves the system throughput in many scenarios. This is achieved by effectively utilizing various beams to provide differentiated services for diverse user distributions. Additionally, the low computational complexity makes it easier to implement in practical applications.

## Figures and Tables

**Table 1:** The set of symbols and their meaning.

Parameter	Symbol	Parameter	Symbol
Beam number	$N_{beam}$	The leaving time of the data packet whose destination user is *i*	$pck^{user_{j}}.{\mathrm{end}}_{time}$
Cell nummber	$N_{cell}$	The delay of one packet.	*D*
delay sensitivity factor	ρ	The total delay of $cell_{i}$.	$D_{cell_{i}}$
Time slot length	$T_{slot}$	The total delay of one period.	Delay
Time slot number	$N_{T}$	The length of the data packet whose destination user is *j*.	$pck^{user_{i}}.len$
Hopping window	*T*	User demand	$D_{user_{j},k}$
The power that is allocated to $cell_{i}$ in *t*.	$P_{cell_{i},t}$	User Rate	$R_{user_{j},k}$
The arrival time of the data packet whose destination user is *i*	$pck^{user_{j}}.{\mathrm{start}}_{time}$	Virtual demand traffic of $cell_{i}$	$VirD_{t}^{cell_{i}}$
The set of service cell	C	Demand traffic of $cell_{i}$	$D_{t}^{cell_{i}}$
The frequency that is allocated to $cell_{i}$ in *t*	$B_{cell_{i},t}$	offered throughput	$R_{t}^{cell_{i}}$
Actual throughput	Throughput		

**Table 2:** The set of parameters and their values.

Parameters	Values
Orbit Height	550 km
Mininum User Elevation	40° (40–90°)
$P_{max}$	1000 w
$G_{R}$	21.8 dBi
$N_{B}$	16
Frequency	2G Hz (19 GHz)
Free Space Loss	20 lg(f) + 20 lg(r) + 32.4 = 169.941664 dB
Noise Temperature	290 K
Boltzmann constant	1.38 × 10^−23^ J/m
Noise Power	KTB = −110.9669292079 (dB)
Delay Sensitivity Factor	2,5 and 10

**Table 3:** Beams size and its gain.

	Radius (km)	Gain (dBi)
Largest size beam	80	31.89
Middle size beam	40	37.89
Smallest size beam	20	43.89

## Data Availability

The original contributions presented in the study are included in the article material, further inquiries can be directed to the corresponding author.
